# Examination of actin and microtubule dependent APC localisations in living mammalian cells

**DOI:** 10.1186/1471-2121-7-3

**Published:** 2006-01-19

**Authors:** Kelly J Langford, Jon M Askham, Tracy Lee, Matthew Adams, Ewan E Morrison

**Affiliations:** 1 CRUK Clinical Centre at Leeds, Division of Cancer Medicine Research, St James's University Hospital, Leeds, LS9 7TF, UK

## Abstract

**Background:**

The trafficking of the adenomatous polyposis coli (APC) tumour suppressor protein in mammalian cells is a perennially controversial topic. Immunostaining evidence for an actin-associated APC localisation at intercellular junctions has been previously presented, though live imaging of mammalian junctional APC has not been documented.

**Results:**

Using live imaging of transfected COS-7 cells we observed intercellular junction-associated pools of GFP-APC in addition to previously documented microtubule-associated GFP-APC and a variety of minor localisations. Although both microtubule and junction-associated populations could co-exist within individual cells, they differed in their subcellular location, dynamic behaviour and sensitivity to cytoskeletal poisons. GFP-APC deletion mutant analysis indicated that a protein truncated immediately after the APC armadillo repeat domain retained the ability to localise to adhesive membranes in transfected cells. Supporting this, we also observed junctional APC immunostaining in cultures of human colorectal cancer cell line that express truncated forms of APC.

**Conclusion:**

Our data indicate that APC can be found in two spatially separate populations at the cell periphery and these populations can co-exist in the same cell. The first localisation is highly dynamic and associated with microtubules near free edges and in cell vertices, while the second is comparatively static and is closely associated with actin at sites of cell-cell contact. Our imaging confirms that human GFP-APC possesses many of the localisations and behaviours previously seen by live imaging of *Xenopus *GFP-APC. However, we report the novel finding that GFP-APC puncta can remain associated with the ends of shrinking microtubules. Deletion analysis indicated that the N-terminal region of the APC protein mediated its junctional localisation, consistent with our observation that truncated APC proteins in colon cancer cell lines are still capable of localising to the cell cortex. This may have implications for the development of colorectal cancer.

## Background

The APC protein plays a pivotal role in WNT signal transduction, has been suggested to have important functions in cell migration and mitosis, and *APC *mutation is a crucial early event in the development of most colorectal cancers [1]. The intracellular localization of APC has long been the subject of close scrutiny, with a number of distributions having been described in a variety of experimental systems (for a recent review see [2]). Two mammalian APC distributions have previously been described as populations found at peripheral cellular sites. The first of these to be identified and widely accepted consists of APC clusters that localize to specific cortical sites in a microtubule-dependent manner [3-5]. Support for this distribution has been presented in studies examining the behavior of *Xenopus *APC-GFP fusion proteins in living cells [6]. In addition, an analogous localization to the plus-ends of microtubules close to the basolateral surface of highly polarized epithelial cells has been shown [7]. Evidence for a second peripheral pool of APC in the form of an actin-dependent localization to membranes involved in cell-cell adhesion has been found by immunostaining studies [3,5] although the validity of this has been questioned [7]. Nevertheless, evidence that potentially supports a functional role for APC at cell junctions has recently been presented. In human cells, restoration of expression of full-length APC in a colorectal cancer cell line has been shown to promote cell-cell adhesion [8] while in *Drosophila *the APC homologue E-APC has been shown to associate with and play a role in maintaining the integrity of epithelial cell junctions, a localization mediated by its armadillo repeats [9].

To date the great majority of information about the distribution of full-length mammalian APC has been based upon immunostaining studies in fixed cells. Since recent work has raised questions about APC antibody specificity [10], confirmation of these different APC distributions by other means is clearly desirable. While some data on GFP-APC expression in COS-7 cells has been previously published [11], this work concentrated on a purely microtubule-associated GFP-APC localization in subconfluent populations of cells and the GFP-APC construct used in the study lacked the N-terminal region of the protein. At present the dynamic behavior of a junctional APC population in live mammalian cells, if such a population exists, has not been addressed.

In this study we investigated the intracellular distribution of APC using live imaging of human GFP-APC fusion proteins in mammalian cells. Our study confirms previous work showing that GFP-APC can be localized within the cell in a microtubule-dependent and highly dynamic manner. However, we show for the first time that human GFP-APC can remain associated with shrinking as well as growing microtubule tips. We also confirm that human GFP-APC is localized to sites of cell-cell adhesion in an actin-dependent way in confluent populations of cells. To our knowledge this is the first time that this actin associated GFP-APC population has been imaged in living mammalian cells.

## Results

### Direct observation of microtubule and cell junction associated GFP-APC in living cells

To date, a junctional association for mammalian APC has only been observed using immunofluorescence microscopy. Immunostaining studies of APC distribution are open to criticisms concerning antibody specificity and fixation artefacts and are unable to yield data about the dynamic behaviour of the protein. Previous attempts to examine this localisation using GFP fusion proteins were hampered by the toxic side effects associated with the expression of full-length APC in mammalian cells [5]. To address this issue and to further define the subcellular distribution of APC we attempted to examine the distribution of a GFP-APC fusion protein in a variety of mammalian cell lines. In most of these transfection efficiencies were low and GFP-APC expression was poorly tolerated. However, our data indicated that COS-7 cells represented a system in which good transfection efficiencies could be achieved and expression levels suitable for imaging were tolerated without obvious short-term effects upon cell morphology or viability. However, since COS-7 cells are not epithelial in origin, we first confirmed that they expressed APC (Figure 2, panel A) and that the endogenous APC in confluent COS-7 cells was localised to the cell cortex along with the known junctional proteins cadherin (Figure 3, panels A-C), β-catenin (Figure 3, panels D-F), and actin (Figure 3, panels G-I). COS-7 cells were found to possess full-length APC protein and this endogenous APC was found to be closely associated with junctional proteins, confirming that these cells do indeed form junctions when confluent. We were therefore confident that we could use this cell line to study dynamic microtubule-associated, as well as junction-associated localisations of our GFP-APC constructs. The integrity of GFP-APC constructs used for localisation studies in COS-7 were confirmed by immunoprecipitation, SDS-PAGE and Western blotting of transfected cell extracts (Figure 2, panel B+C). The largest of these fusion proteins (Figure 2, panel B, lanes 1–3), consisting of full-length GFP-APC and minimal deletion mutants, are indistinguishable by band locations on Western blots with the gel and blotting systems used in this study because of their large size.

**Figure 1:**
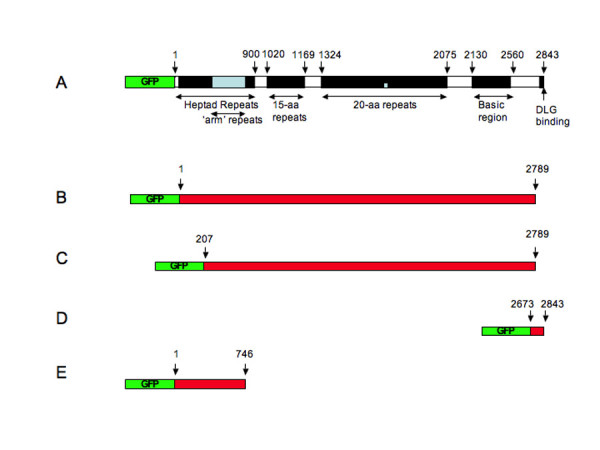
**Schematic of full-length and deletion GFP-APC constructs used in this study (not to scale)**. Panel A. Full-length GFP-APC including known APC domains. Panel B. pEGFP-APCΔC construct lacking the last 54 amino acids of APC. Panel C. pEGFP-APCΔN+ΔC construct lacking the first 206 and last 54 amino acids of APC. Panel D. pEGFP-APC-C construct consisting of the last 170 amino acids of APC. Panel E. pEGFP-APC-N consisting of the first 746 amino acids of APC.

**Figure 2:**
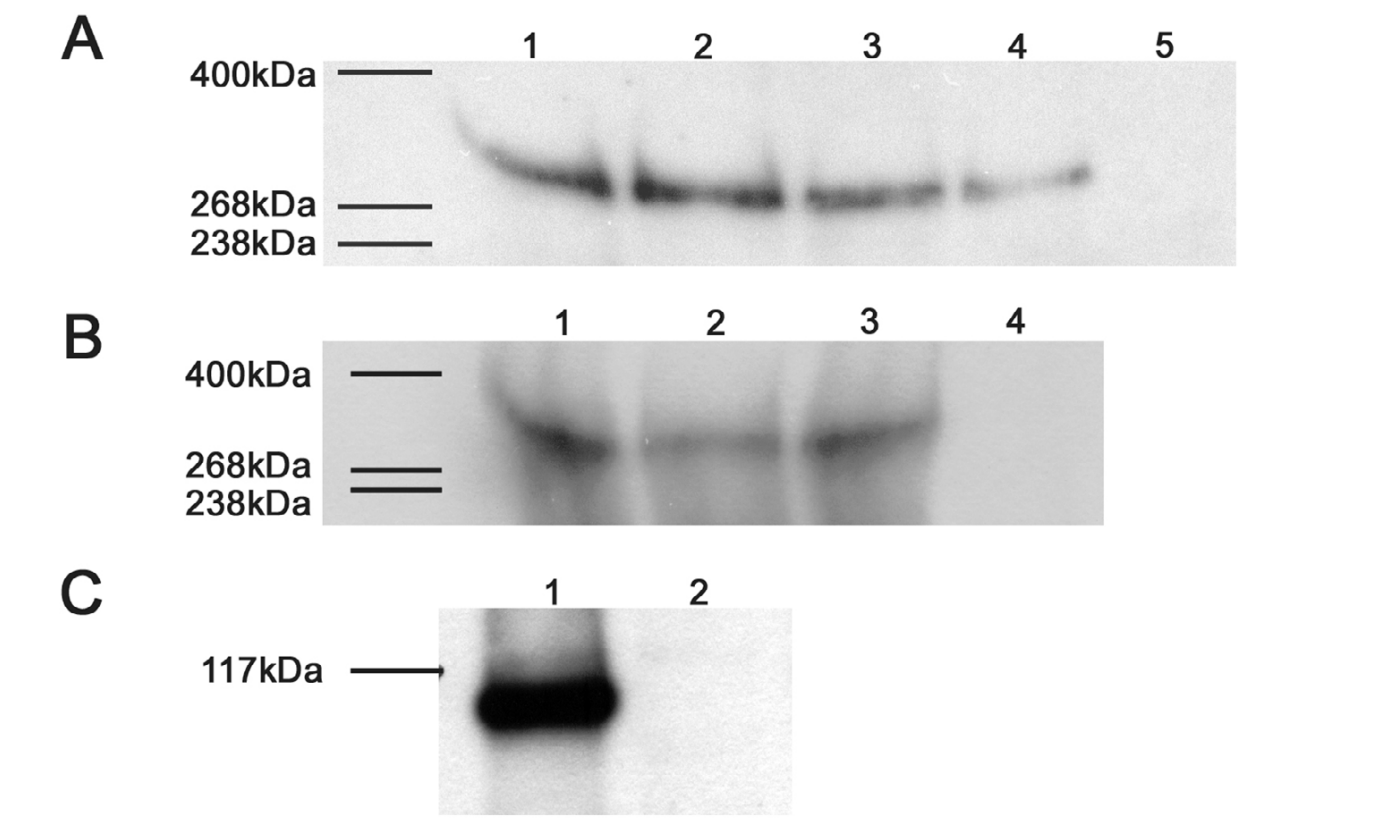
**Western blotting of endogenous APC and GFP-APC constructs expressed in COS-7 cells**. Panel A: Western blot of cell extracts from COS-7 (lane 1), HeLa (lane 2), MDCK (lane 3), NRK-52E (lane 4) and Caco-2 (lane 5), 10 μg total protein was loaded for each extract and the membrane probed with the ALI 12-28 antibody specific for the APC N-terminus. Full-length APC (310 kDa) can be detected in all cell lines except CaCo-2 cells that possess a much smaller truncated APC protein (not shown). Panel B: Western blot of COS-7 cell extracts transfected with plasmids directing the expression of GFP-APC (lane 1), GFP-APC-ΔN (lane 2), GFP-APC-ΔN+ΔC (lane 3), and untransfected COS-7 cells (lane 4). Prior to western blotting extracts were immunoprecipitated with a polyclonal anti-GFP antibody. The immunoprecipitation from each extract was loaded onto a gradient gel, subjected to SDS-PAGE, transferred to nitrocellulose and the membrane probed with a polyclonal anti-GFP antibody. Panel C: Western blot of the COS-7 cell extract transfected with the plasmid directing the expression of GFP-APC-N construct (lane 1) or a non-transfected COS-7 cell extract (lane 2). 20 μg of each protein extract was loaded onto a 10% non-gradient gel and the membrane probed with a polyclonal anti-GFP antibody.

**Figure 3:**
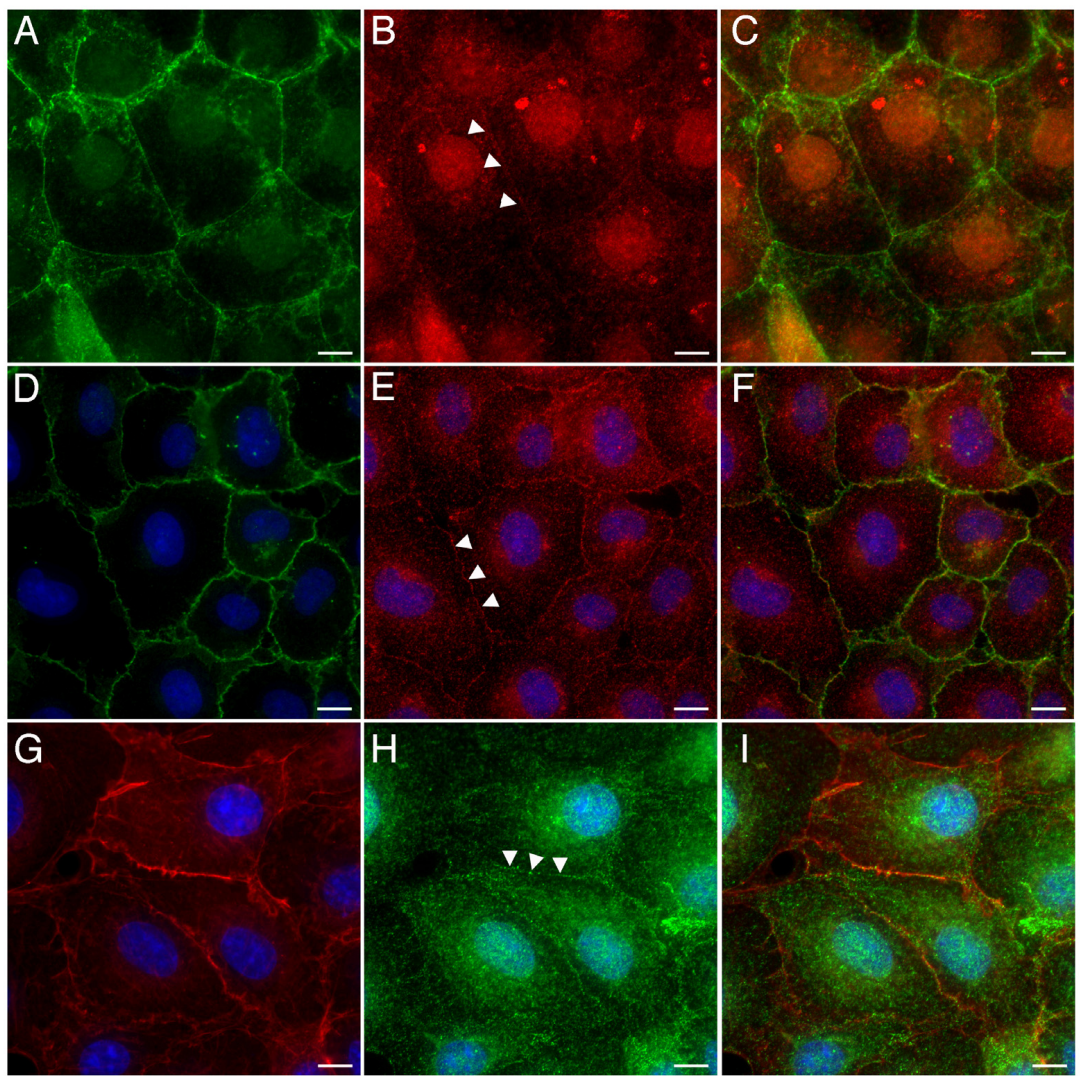
**Junction formation and APC localisation in COS-7 cells**. Panels A-C. COS-7 cells contain cadherins and endogenous APC (arrowheads) at sites of cell-cell contact, Cadherin green and APC red, panel C is a merged image. Panels D-F. COS-7 cells also have the junctional protein β-catenin localised along with endogenous APC (arrowheads) at adhesive membranes. β-catenin green, APC red and DAPI blue, panel F is a merged image. Panels G-I. Cortical actin and APC (arrowheads) are associated with sites of cell-cell contact in COS-7 cells. Actin red, APC green and DAPI blue, Panel I is a merged image. Bars = 10 μm.

**Figure 4:**
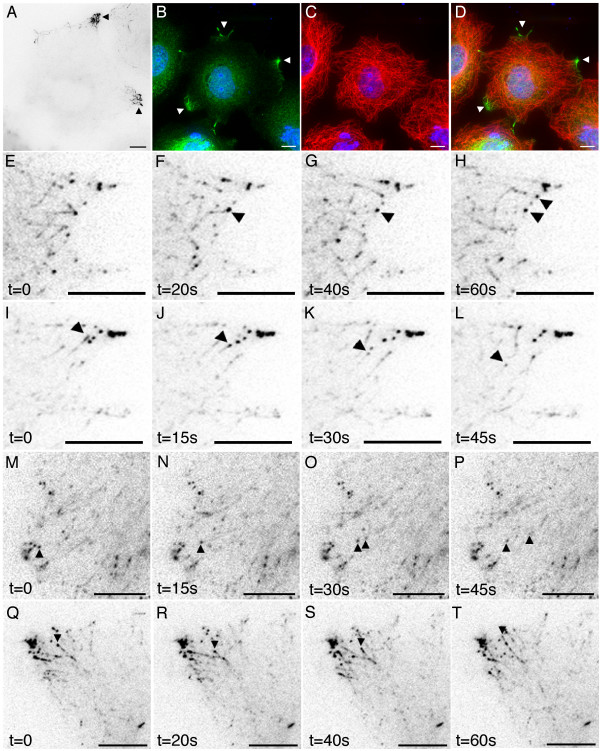
**Detailed imaging of full-length GFP-APC in living COS-7 cells**. Panel A. In subconfluent cells GFP-APC decorates the distal tips of microtubules at cell vertices (arrows, additional file 1, 5s time lapse). Panels B-D. GFP-APC clusters decorating distal tips of microtubules in fixed GFP-APC expressing cells. GFP-APC green, tubulin red and DAPI blue, panel D is a merged image. Panels E-H. GFP-APC puncta can be deposited at the cell cortex (arrows, additional file 2). Panels I-L. GFP-APC puncta could be seen undergoing retrograde movements at the cell periphery (arrowhead, additional file 3). Panels M-P. Part of an original GFP-APC puncta is lost from a shrinking tip while the remaining attached portion continues retrograde movement (arrowheads, additional file 4). Panels Q-T. Some shrinking microtubules tipped with GFP-APC puncta undergo re-growth after periods of shrinkage (arrowhead, move 5). Bars = 10 μm.

A variety of GFP-APC distributions could be observed in COS-7 cells using time-lapse fluorescence microscopy, all consistent with either previous immunostaining studies or known interactions of mammalian APC, suggesting that our GFP-APC imaging was a good reflection of endogenous APC behaviour. The first was an association with microtubule tips at specific regions near the cell periphery (Figure 4, panel A (arrowheads); additional file 1). This consisted of labelling of microtubule distal segments and the presence of discrete GFP-APC puncta or clusters on or just behind the microtubule tip. Cells expressing GFP-APC were co-stained with anti-α-tubulin, confirming that the GFP-APC clusters were indeed microtubule associated (Figure 4, panels B-D). GFP-APC puncta on the end of these microtubules were observed to move at speeds consistent with microtubule growth (18 ± 8.4 μm/min; n = 28 puncta from 3 cells where movement could be continuously observed for at least 12s). As previously shown by workers using *Xenopus *GFP-APC, GFP-APC puncta located behind the microtubule tip underwent anterograde movement towards the tip [6]. Peripheral deposition of GFP-APC puncta by shrinking microtubules could also be observed (Figure 4, panels E-H; additional file 2). These observations closely resembled both previous reports of APC distribution based upon immunostaining (e.g. [3-5] and the APC-GFP distribution and behaviours described in *Xenopus *cells [6]).

However, we noted in COS-7 cells that peripheral GFP-APC puncta on microtubule ends frequently underwent retrograde linear movements with average velocities of 21.6 ± 2.4 μm/min (Figure 4, panels I-L (arrowhead); additional file 3). Typically this movement occurred over distances of less than 10 μM. In some cases portions of an original puncta were deposited within the cytoplasm while the remainder continued retrograde movement on the microtubule end (Figure 4, panels M-P arrowheads; additional file 4, examples also apparent in additional file 3). We also noted that the depolymerising microtubules tipped by GFP-APC puncta could undergo pause and then re-growth with the puncta of GFP-APC still attached (Figure 4, panels Q-T arrowhead; additional file 5). Interestingly, close examination of sequences such as additional file 5 indicates that separate GFP-APC puncta decorating the distal segment of a microtubule are swept up into a single tip-associated structure as the microtubule shrinks but re-separate into a string of beads-like distribution during microtubule regrowth, before finally beginning to re-coalesce into a single structure at the tip of paused microtubules near the cell membrane. The behaviour of GFP-APC at microtubule distal tips is clearly therefore very complex. Retrograde trafficking of GFP-APC was not documented in previous studies [6,11]. Furthermore, these movements seem likely to represent puncta associated with shrinking microtubule tips and not with growing tips looping back from the cell edge or retrograde transport of GFP-APC puncta along microtubules since the microtubule distal segment could clearly be identified by GFP-APC labelling in many cases. We therefore conclude that mammalian APC can remain associated with shrinking microtubule tips at the cell periphery, a novel observation for this protein.

**Figure 5:**
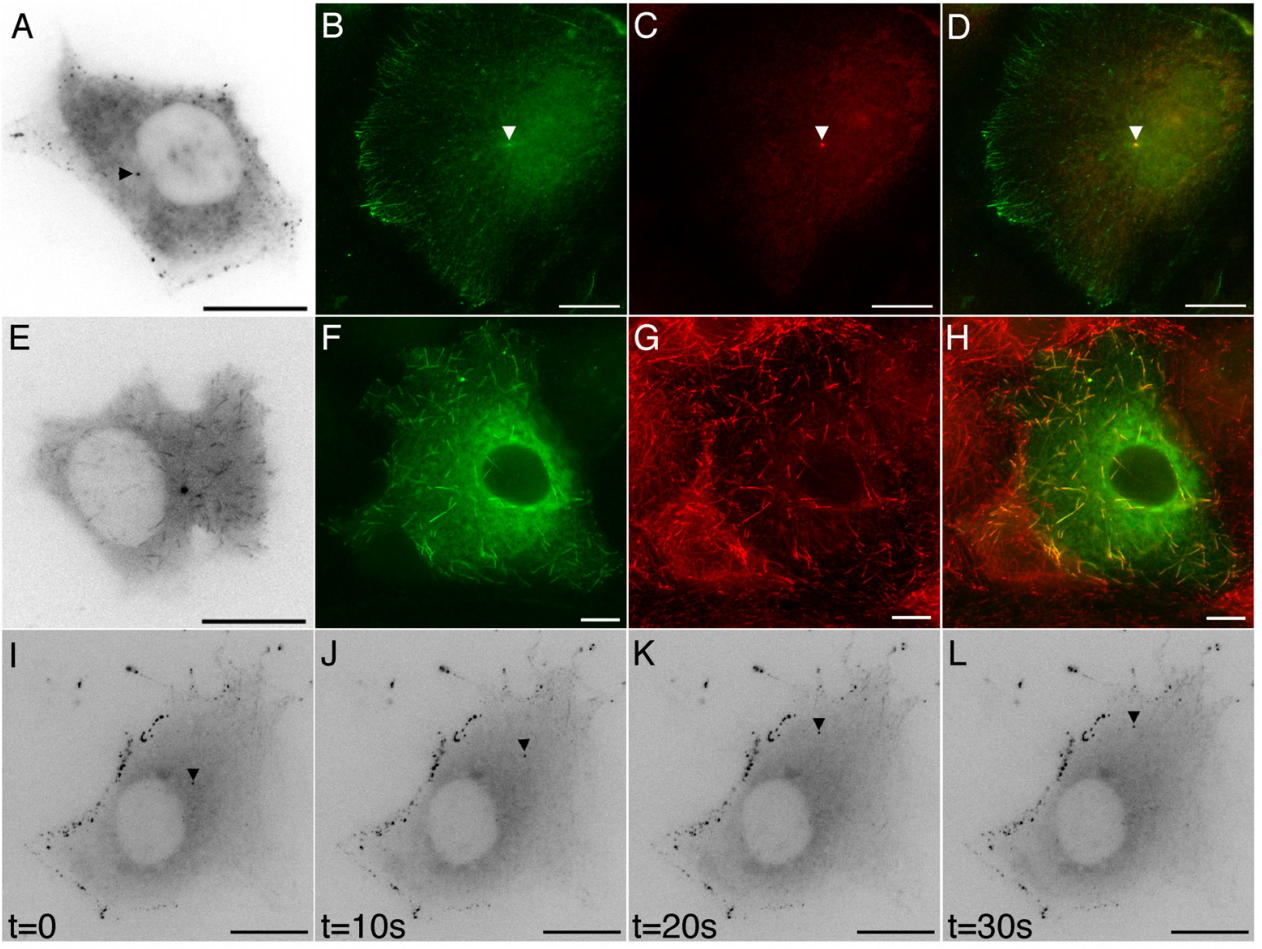
**Additional localisations seen in GFP-APC expressing COS-7 cells**. Panel A. In some cells GFP-APC can be seen at the centrosome (arrowhead). Panels B-D. Localisation of GFP-APC to the centrosome was confirmed in immunostained cells using the centrosomal marker γ-tubulin. GFP-APC green, γ-tubulin red, panel D is a merged image. Panel E. A minor population of transfected cells had a comet-like GFP-APC distribution (additional file 6). Panels F- H. Co-staining of GFP-APC expressing COS-7 cells with EB1 confirmed the two proteins co-localise at growing microtubule tips in some cells. GFP-APC green, EB1 red, Panel H is a merged image. Panels I-L. Occasional cells contained GFP-APC puncta that were rapidly transported through the cytoplasm (arrowhead, additional file 7). Bars = 10 μm

These observations represented the major GFP-APC distribution in cells imaged in subconfluent cultures although other minor localisations were also seen. GFP-APC was occasionally observed at structures resembling the centrosome (Figure 5, panel A, arrowhead). This localisation was confirmed in fixed COS-7 cells expressing GFP-APC co-stained with γ-tubulin (Figure 5, panels B-D). A centrosomal localisation for APC was recently examined in detail by other investigators [12]. In less than 5% of cells a motile comet-like distribution within the cell interior was seen (Figure 5, panel E; additional file 6). These comets moved with an average velocity of 20.4 ± 6 μm/min (n = 29 comets from 3 cells where movement could be tracked for at least 12s). This was essentially indistinguishable from the distribution and behaviour observed for the microtubule tip-tracking APC ligands EB1 and EB3 when expressed as GFP fusion proteins in COS-7 cells (JMA, unpublished data). GFP-APC expressing COS-7 cells fixed and co-stained for EB1 showed that the GFP-APC comets did indeed co-localise with EB1 staining at the end of microtubules (Figure 5, panels F-H). Finally, GFP-APC puncta were occasionally observed in the cell interior (Figure 5, panel I-L; additional file 7). Over the time course of imaging these were either immobile or underwent rapid translocations at peak speeds consistent with microtubule motor-mediated transport (in the region of 2–3 μm/s). Previous reports have described an interaction between APC and kinesin-associated transport complexes [13] that could explain this behaviour.

**Figure 6:**
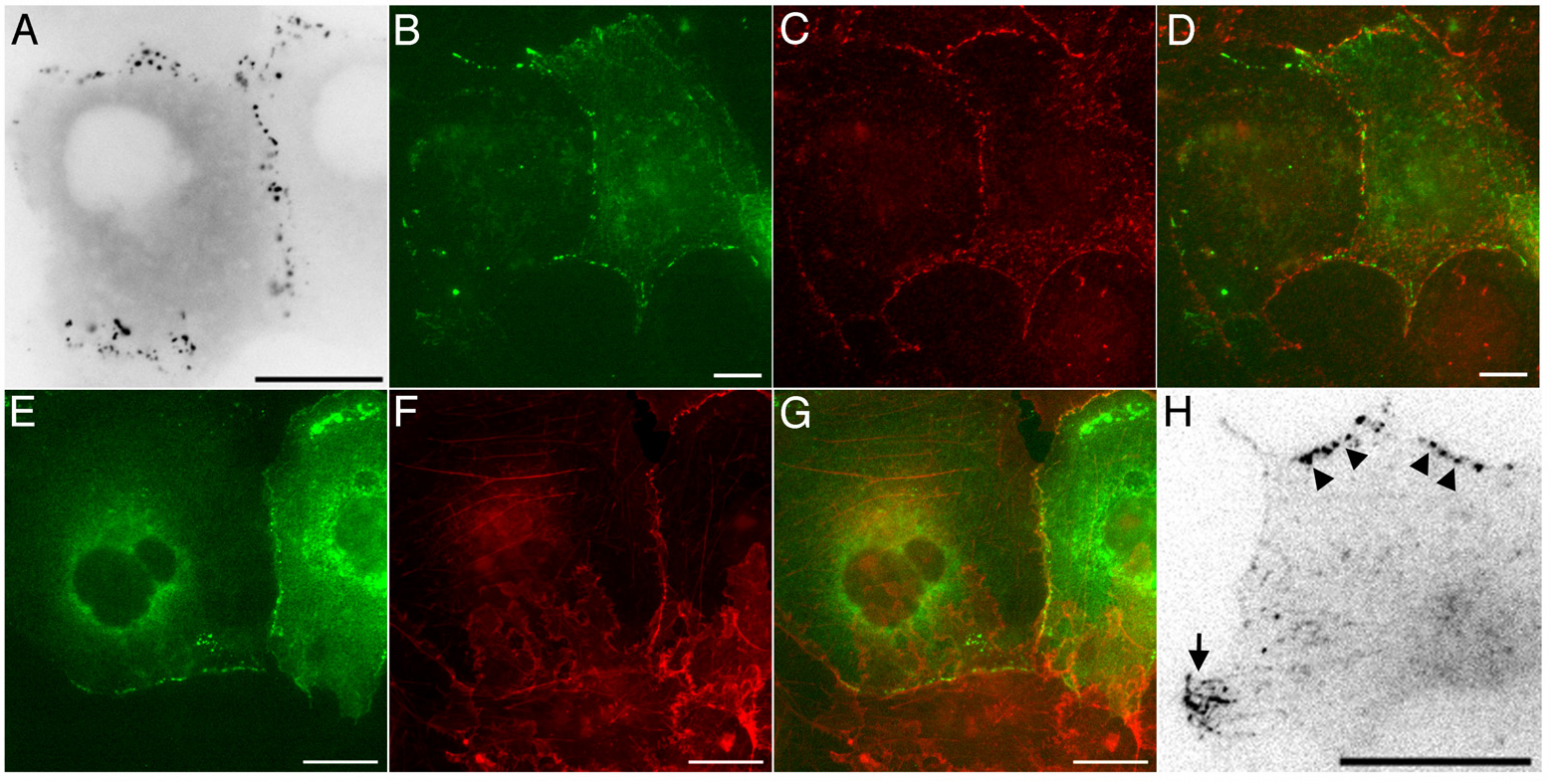
**Localisation of GFP-APC to junctional membranes in COS-7 cells**. Panel A. In highly confluent cells GFP-APC showed a purely junctional localisation (additional file 8,). Panels B-D. GFP-APC at the cortex in COS-7 cells is located close to the junctional protein α-catenin. GFP-APC green, α-catenin red, panel D is a merged image. Panels E-G. GFP-APC is also closely associated with cortical actin at sites of cell-cell adhesion. GFP-APC green, actin red, Panel G is a merged image. Panel H. Both junctional (arrowheads) and microtubule-associated (arrow) GFP-APC populations can co-exist in the same cell (additional file 9). Bars = 10 μm.

In cultures that were seeded and transfected to be largely confluent during imaging a further GFP-APC distribution was seen. This consisted of a discontinuous array of punctate structures associated with peripheral membranes contacting other cells (Figure 6, panel A; additional file 8). This junctional localisation was confirmed by co-immunostaining for β-catenin (Figure 6, panels B-D) and actin (Figure 6, panels E-G), both of which confirmed that GFP-APC was closely associated (but not co-localised) with junctional proteins at the cortex. In comparison with the microtubule-associated GFP-APC distributions these cortical structures were far less dynamic. Tracking analysis indicated that they moved with an average velocity of 0.3 ± 0.36 μm/min (n = 70 puncta from 4 cells continuously observed for at least 10 min). This movement appeared to directly correlate with remodelling of the cell periphery during long-term changes in cell shape, indicating that these structures were essentially immobile relative to the cell cortex.

The junctional GFP-APC localisation was dominant in cells having extensive contacts with neighbouring cells, particularly those in densely confluent regions of an imaging dish. However, in regions of lower cell density we observed that both the microtubule and cell junction-associated GFP-APC distributions could be found in different regions of the same cell (Figure 6, panel H; additional file 9). The junctional population seen in these cells was unlikely to represent an overexpression artefact since it was present at low GFP-APC fluorescence intensities and no phenomena indicative of APC overexpression were seen (for example, GFP-APC decorated microtubule bundles). Observation of cells with both junctional- and microtubule-associated GFP-APC confirmed the very different dynamic behaviours of these protein populations. The microtubule-associated GFP-APC localisation was restricted to free cell edges and cell vertices and was highly motile whereas the junctional localisation was only present at sites of cell-cell contact and was essentially immobile. Dynamic GFP-APC microtubule-associated clusters were not seen in the vicinity of cell junctions, although when present the EB1-like localisation to growing microtubule distal tips was. We therefore suggest that the two GFP-APC populations might reflect the localised regulation of APC interactions in specific cellular regions.

**Figure 7:**
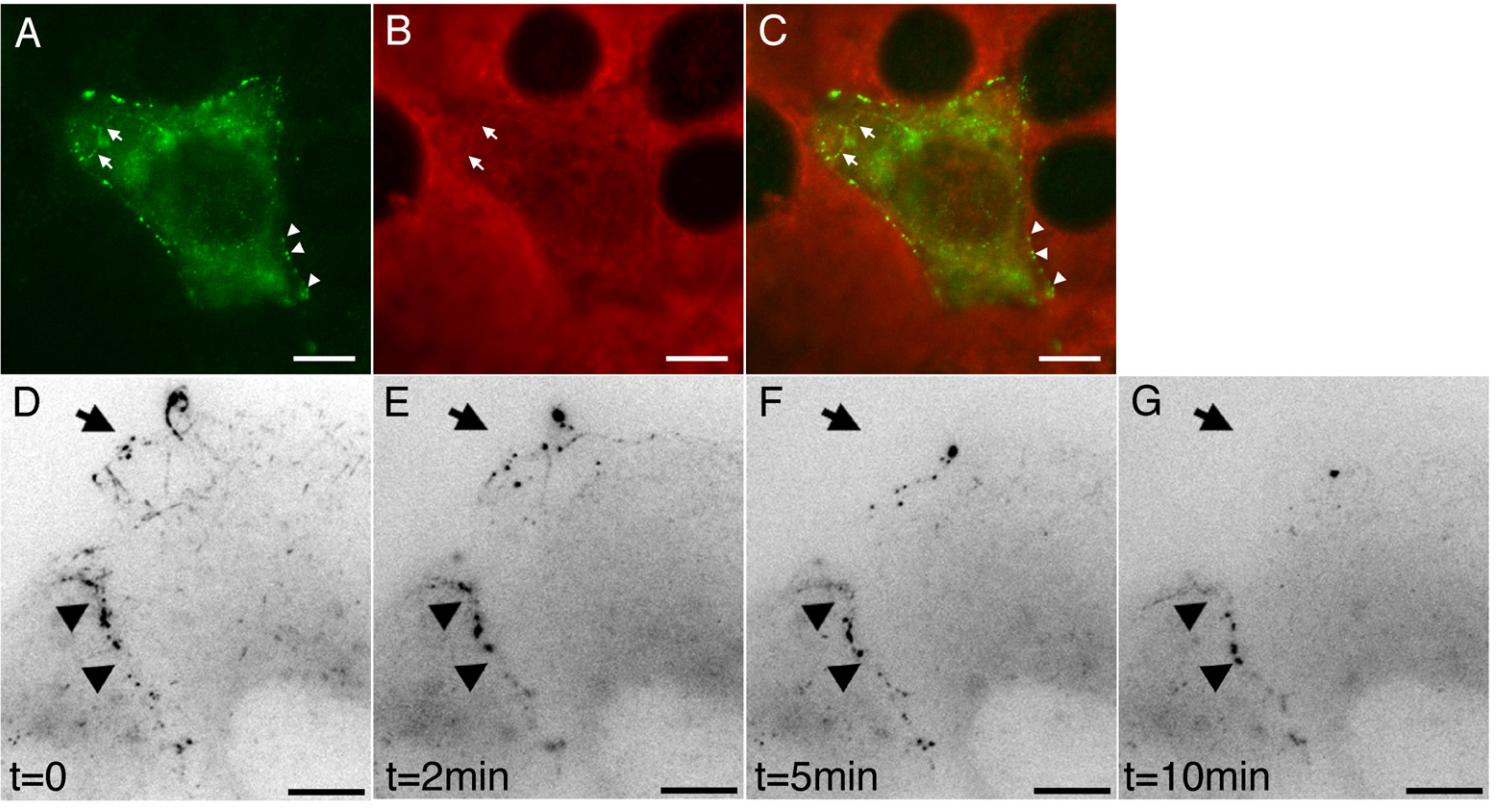
**Effects of microtubule depolymerisation on GFP-APC distribution in COS-7 cells**. Panels A-C. GFP-APC expressing COS-7 cells were incubated for 60 min in 5 μg/ml Nocodazole. GFP-APC associated with remnant microtubules (arrows) can be found in Nocodazole treated cells. However, junctional GFP-APC (arrowheads) is not associated with microtubules and appears unaffected by Nocodazole treatment. GFP-APC green, tubulin red, panel C is a merged image. Panels D-G. Nocodazole treatment of living cells possessing microtubule-associated and junctional-associated GFP-APC (additional file 10). Microtubule-associated GFP-APC (arrow) disperses over time but junctional GFP-APC puncta (arrowheads) are unaffected.

**Figure 8:**
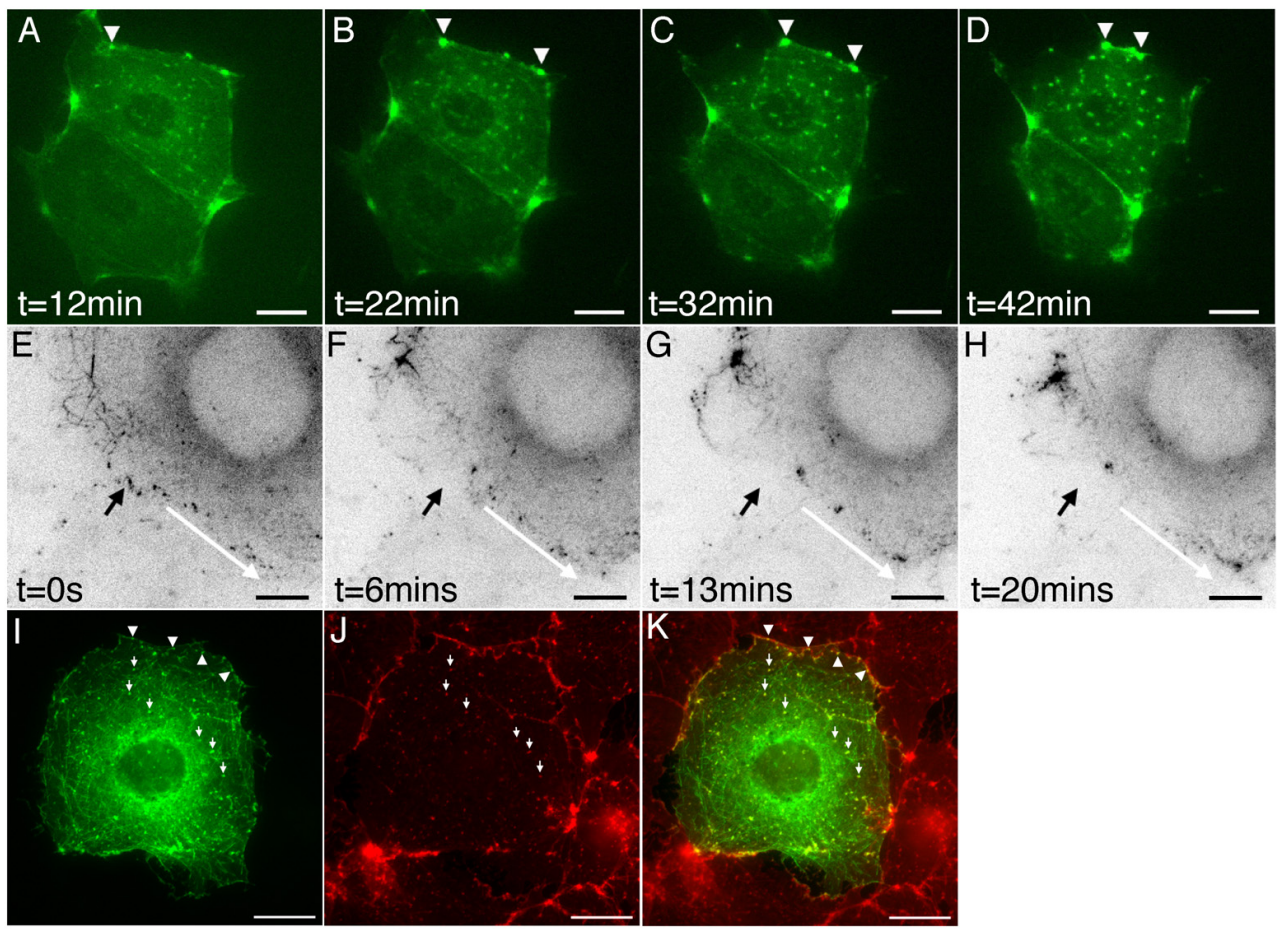
**Effect of actin poison on GFP-APC localisation in COS-7 cells**. Panels A-D. Treatment of GFP-actin expressing COS-7 cells with 1 μg/ml Cytochalasin D gradually perturbed the integrity of the cortical actin ribbon. With increasing time of exposure to drug the cortical actin ribbon thins, snaps and contracts (arrowheads, additional file 11 arrows). Bars = 20 μm. Panels E-H. Cytochalasin D treatment (1 μg/ml) of cells possessing both microtubule-associated and junctional GFP-APC (additional file 12). Following drug addition cell-cell contacts decorated with GFP-APC puncta can be seen to break (black arrow), followed by movement of the GFP-APC puncta along the cell edge towards cell vertices (white arrow shows direction of movement). The dynamic behaviour of microtubule-associated GFP-APC continues within the constraints imposed by retraction of the free cell edge. Bars = 10 μm. Panels I-K. Paraformaldehyde fixed, phalloidin-stained cells expressing GFP-APC after 30 min treatment with Cytochalasin D (1 μg/ml). Co-localization of GFP-APC with actin aggregates in the cytoplasm (arrows) and with cortical actin (arrowheads) is seen. GFP green, phalloidin red, panel K is a merged image. Bars = 20 μm.

Previous reports have shown that microtubules can be resistant to Nocodazole treatment in cells overexpressing microtubule-associated GFP-APC [14]. In order to test the effect of Nocodazole on transfected COS-7 cells possessing cortical and microtubule-associated GFP-APC we first incubated cells with 5 μg/ml Nocodazole for 1 hour (Figure 7, panels A-C). We noted that even after a one-hour incubation cells containing a small number of stable microtubules could be found (Figure 7, panel B, arrows). In transfected cells these remnant microtubules were decorated with GFP-APC (Figure 7, panels A-C). However, cortical APC could be clearly distinguished at the cell cortex in the absence of microtubules and appeared unaffected by Nocodazole treatment (Figure 7, panels A-C, arrowheads). We next examined the effects of microtubule depolymerisation on GFP-APC-cortical localisations in living cells. Figure 7, panels D-G and additional file 10 shows a cell in which both junctional (Figure 7, panels D-G arrowheads, also see additional file 10) and microtubule (Figure 7, panels D-G arrow, also see additional file 10) GFP-APC populations are present. At the beginning of the recording Nocodazole was added to a final concentration of 5 μg/ml. As previously reported by other investigators [6], the GFP-APC localization to microtubule distal tips was rapidly lost and the cortical clusters at microtubule ends gradually dispersed. However, the behaviour and intensity of junctional GFP-APC was unaffected by Nocodazole, even in subsequent experiments where the drug concentration was raised to 20 μg/ml for 30 min (data not shown). We conclude that the junctional association of GFP-APC is independent of microtubules. We next wanted to study the response of junctional GFP-APC to treatment with the actin depolymerising drug Cytochalasin D. Preliminary experiments with this drug at standard experimental concentrations of 10 μg/ml led to rapid cell rounding (data not shown). This made it impossible to study the effects of Cytochalasin D on cortical GFP-APC dynamics in these cells. We therefore decided to use low concentrations (1 μg/ml) of drug in order to study the effect of gradual, but not total, loss of cortical actin in COS-7 cells. Treatment of COS-7 cells expressing a GFP-actin construct [15] confirmed that at low concentrations Cytochalasin D perturbed actin integrity at the cell cortex in a stereotypical way. Figure 8, panels A-D, and additional file 11 show a GFP-actin expressing COS-7 cell treated with 1 μg/ml Cytochalasin D. At the beginning of the experiment the cell possesses a cortical actin belt around the cell periphery. Over the time course of the experiment this cortical belt can be seen to weaken and break at points within the cell (Figure 8, panels A-D arrowheads and additional file 11), These breakages lead to the contraction of the cortical actin belt along cell edges and the accumulation of actin at cell vertices. We next examined the results of low-level Cytochalasin D treatment in GFP-APC expressing cells. The results of a typical experiment are shown in Figure 8, panels E-H, and additional file 12. The cell of interest in this sequence has both cortical- and microtubule-associated GFP-APC. The earliest response, beginning around 10 min after drug addition, was a slow contraction of the whole cell; this was followed by breakage of the cell-cell contacts (Figure 8, panels E-H black arrow, and additional file 12) and the contraction of the GFP-APC puncta along the cell edge (Figure 8, panels E-H white arrow indicates direction of contraction, and additional file 12). This data closely resembled the effects of Cytochalasin D on the junction-associated cortical actin ribbon in COS-7 cells expressing GFP-actin (Figure 8, panels A-D, and additional file 11). When confluent GFP-APC expressing COS-7 cells were treated with 1 μg/ml Cytochalasin D for 30 min before fixation and staining with phalloidin an association of GFP-APC with both cytoplasmic actin aggregates (Figure 8, panels I-K, arrows) and cortical actin (arrowheads) was seen, confirming that the distribution of GFP-APC was closely linked to that of actin in these cells. These drug experiments lead us to conclude that in transfected COS-7 cells two spatially separate populations of GFP-APC exist at the cell periphery and can co-exist in the same cell. The first is highly dynamic and associated with microtubules near free edges and cell vertices, while the second is comparatively static and is closely associated with actin at sites of cell-cell contact.

### Deletion analysis of the APC-cell junction association

**Figure 9:**
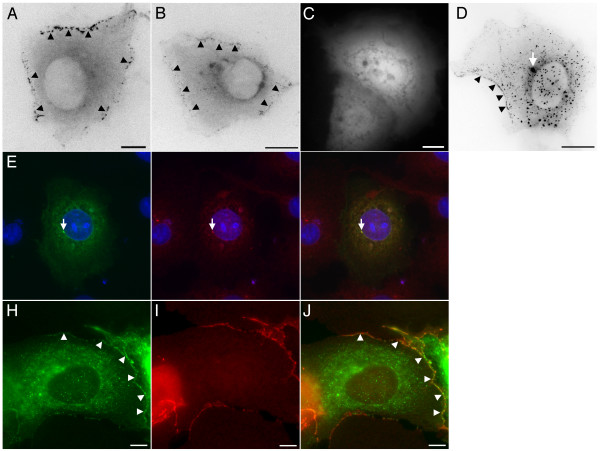
**Distribution of truncated APC proteins**. Panels A and B. GFP-APCΔC and GFP-APCΔNΔC localize to both the cortex (arrowheads) and to microtubules (data not shown) in a manner similar to that previously seen for GFP-APC. Panel C. The short *C*-terminal fragment GFP-APC-C1 has a cytoplasmic localization similar to that seen with GFP alone (data not shown). Panel D. GFP-APC-N localizes to the centrosome (arrow), cytoplasmic puncta and the cortex (arrowheads, additional file 13). Panels E-G. GFP-APC-N expressing cells co-immunostained for the centrosomal marker γ-tubulin show GFP-APC-N localises to the centrosome (arrow). GFP-APC-N green, γ-tubulin red, panel G is a merged image. Panels H-J. GFP-APC-N expressing cells co-immunostained for β-catenin confirm the GFP-APC-N junctional localisation (arrowheads). GFP-APC-N green, β-catenin red. Panel J is a merged image. Bars = 10 μm.

To further define the domain in APC responsible for mediating its junctional localisation we made a number of GFP-APC deletion constructs for analysis in COS-7 cells. It seemed possible that the junctional localisation of APC cells could be mediated by an interaction with the junctional protein Discs Large (DLG), a known ligand of APC [16]. This interaction requires the extreme *C*-terminus of the APC molecule. We therefore made a construct, pEGFP-APCΔC (Figure 1, panel B), which directed the expression of full-length GFP-APC lacking its final 54aa. APC also dimerises using heptad repeats at the *N*-terminus of the protein and deletion of the first 58 amino acids of APC prevents this [17,18]. Since any construct made by us might potentially be able to localize to junctions by interacting with endogenous APC, we therefore made a second construct, pEGFP-APCΔNΔC (Figure 1, panel C). This directed the expression of GFP-APC lacking both its final 54aa and first 207aa, which would both abolish DLG binding and prevent dimerisation. Expression of these constructs in transfected cells was confirmed by western blotting (Figure 2, panel B). In transfected COS-7 cells examined by time-lapse fluorescence microscopy the distribution of both of these fusion proteins closely resembled that of full-length GFP-APC, with the full range of both microtubule associated (not shown) and junctional APC localisations observed (Figure 9, panels A and B, arrowheads). This indicated that DLG binding was not required to recruit APC to the cortex in COS-7 cells and suggested that APC dimerisation was also unnecessary. A third construct, pEGFP-APC-C1 (Figure 1, panel D), drives the expression of a protein consisting of the last 170 amino acids of APC fused *N*-terminally to EGFP and would be predicted to interact with DLG. It showed a diffuse cytoplasmic localization within transfected cells (Figure 9, panel C), suggesting that a DLG interaction alone is insufficient for APC targeting to junctions in COS-7 cells. The final construct we examined, pEGFP-APC-N (Figure 1, panel E), directed the expression of a GFP-APC molecule truncated just after its armadillo repeat motifs. This construct showed three distinct localizations within the cell. The first was to the centrosome (Figure 9, panel D, arrow), confirmed by co-immunostaining of transfected cells for the centrosomal marker γ-tubulin (Figure 9, panels E-G). This observation confirms previous studies indicating a centrosomal localization for truncated APC proteins [19]. The second localization was to small, sometimes motile puncta within the cytoplasm (Figure 9, panel D; additional file 13). As noted above, the armadillo repeat region of APC has been shown to mediate an association with kinesin [13]. This association may potentially mediate this localization, but confirming this hypothesis was beyond the scope of the present study. The third localization was to sites of cell-cell contact in a distribution that resembled that previously seen with the full-length GFP-APC protein (Figure 9, panel D, arrowheads; additional file 13), confirmed by co-immunostaining for β-catenin (Figure 9, panels H-J. These data strongly suggest that the APC localization to cell junctions is mediated by a domain lying within the first 746aa of the protein. Unlike our longer GFP-APC fusion proteins, GFP-APC-N did not localise in dynamic clusters or decorate microtubule distal tips in specific regions of the cell periphery, nor did it localise to growing microtubule ends in the cell interior, consistent with a role for the APC microtubule and EB protein binding domains in mediating these localisations.

**Figure 10:**
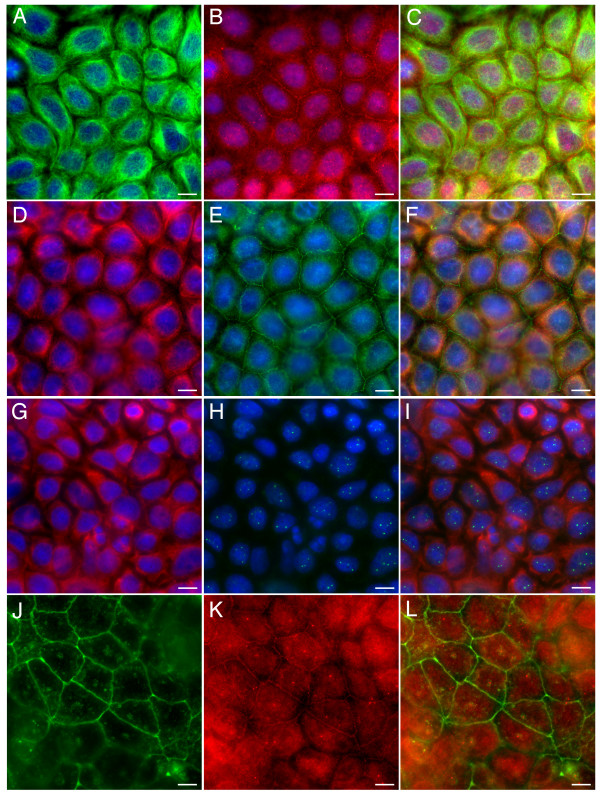
**Junctional staining of the colon cancer cell line Caco-2**. Panels A-C. Confluent Caco-2 cells were co-immunostained with the polyclonal M-APC antibody and for microtubules. A microtubule-independent junctional localisation is seen. Tubulin green, APC red and DAPI blue. Panel C is a merged image. Panels D-F. Confluent Caco-2 cells immunostained using the monoclonal antibody ALI 12-28. Tubulin red, APC green and DAPI blue. Panel F is a merged image. Panels G-I. A monoclonal antibody specific for the APC C-terminus shows no specific APC localisation, as would be expected with the truncated APC expressed in Caco-2 cells. Tubulin red, APC green and DAPI blue, panel I is a merged image. Panels J-L. Actin and APC co-staining in Caco-2 cells confirms the localisation of APC to junctional membranes. Actin green, M-APC red, panel L is a merged image. Bars = 10 μm.

Our data indicated that *C*-terminally truncated GFP-APC molecules retained an ability to associate with sites of cell-cell contact in transfected cells. The pEGFP-APC-N (Figure 1, panel E) construct used in this study is similar to some of the more extremely truncated APC proteins expressed in colorectal cancer cells. Our observations might therefore predict that mutant APC should also localise to sites of cell-cell adhesion in cancer cells, as long as these cells were capable of assembling functional intercellular junctions. We therefore looked for colon cancer cell lines containing similar truncations to our pGFP-APC-N construct. The cell line COLO320 expresses an APC protein truncated at amino acid 811. Unfortunately however, this cell line displays very poor cell-cell adhesion so it was not possible to look at an APC junctional localisation in this cell type. We therefore examined the distribution of endogenous APC in the human colorectal tumour cell lines Caco-2 and SW480. Caco-2 cells possess one mutant copy of APC and express a protein truncated at amino acid 1367. These cells form adhesive junctions and although the truncated APC protein is longer than our minimal construct the question of whether C-terminally truncated APC proteins could localise to the cortex could still be addressed. The polyclonal M-APC antibody and the monoclonal antibody ALI12-28 would both be predicted to recognise the truncated APC protein. Unlike a previous report [5], we found that truncated APC localized to sites of cell-cell adhesion in Caco-2 cells following immunostaining with the polyclonal M-APC antibody (Figure 10, panels A-C) or the monoclonal ALI-12-28 antibody (Figure 10, panels D-F), but not with monoclonal antibodies specific for the APC C-terminus (Figure 10, panels G-I). Cells were also co-stained for actin to confirm the presence of adhesive junctions in the confluent cultures (Figure 10, panels J-L). The colon cancer cell line SW480, containing an APC protein truncated at amino acid 1338, produced similar results to those seen with Caco-2 cells (data not shown).

## Discussion

In this study we have confirmed that APC can be found at sites of cell-cell contact in mammalian cells by live imaging of GFP-APC fusion proteins. Recently, other workers have presented an examination of GFP-APC dynamic behaviour in transfected COS-7 cells [11] but did not report findings similar to those presented here. The reasons for this are unclear. Although the GFP-APC construct used in the previous study contained a short *N*-terminal deletion our data indicates that this would not have precluded the observation of a junctional APC population (Figure 9, panel B). However, we note that the authors of this previous study were primarily focused upon defining the behaviour of GFP-APC decorated microtubules at peripheral sites rather than examining the possibility of a GFP-APC pool at cell junctions. They may not therefore have examined GFP-APC expressing cells in confluent cell cultures. Similarly, we are unable to conclusively explain why previous attempts at mapping the minimal region required for the GFP-APC junctional localisation were unsuccessful and led to the hypothesis that only full-length APC could achieve this distribution [5]. We suspect, however, that difficulties similar to those we experienced in attempting to image GFP-APC in cell types other than COS-7 were a major contributory factor to this.

In addition to the microtubule-associated GFP-APC localisations found by other workers we identified an association of GFP-APC puncta with shrinking microtubule tips in transfected COS-7 cells (Figure 4, panels I-L and panels M-P). APC is known to promote microtubule stability and assembly both in vitro and in vivo [20-22] and as such we might have expected it to be found only on polymerising microtubule tips. The presence of GFP-APC puncta on depolymerising tips would suggest that additional proteins involved in microtubule dynamics might regulate the effects of APC on microtubule plus-end dynamics. For example, the presence of a destabilizing factor on a microtubule tip, such as the kin I kinesin MCAK [23,24], might override the normal stabilising ability of APC. However, if the destabilising factor is inactivated on the microtubule, any tip-associated APC could then re-promote microtubule stability and growth. That *Xenopus *APC has recently been shown to associate with MCAK raises the interesting possibility that APC might form part of a microtubule plus-end complex responsible for the general control of microtubule behaviour in specific regions of the cell periphery [25].

The mechanism by which APC associates with actin at cell junctions remains unclear. An interaction between APC and the mammalian homologue of the *Drosophila *discs large protein has been proposed to be responsible for the localization of APC to neuronal synapses [16], structures that can be regarded as a specialised form of cadherin-based cell-cell adhesion. However, we found that this interaction was not essential for localising APC to sites of cell-cell contact in COS-7 cells although an interaction between APC and DLG at the cortex once both proteins have been recruited there cannot be ruled out. Instead, our data indicated that a truncated APC containing only the heptad and armadillo repeat domains of the protein was capable of localizing to junctions.

We therefore propose that the N-terminal region of the APC protein mediates its localization to the cortex. We speculate that the armadillo repeat regions of APC mediate this localisation since we also noted that a larger construct lacking a number of the heptad repeats remained capable of localising to adhesive membranes (Figure 9, panel B). However, further work needs to be carried out to directly confirm this. This may be complicated by the observation that a number of potential binding partners exist for the N-terminal region of APC, particularly the armadillo repeat domain. These include the kinesin-associated protein KAP3, the Rac effector Asef and the Cdc42 activator IQGAP1 [13,26,27]. Any of these could plausibly mediate an APC junctional localisation, as could an interaction with an as yet unidentified binding partner for this region of APC. Notably, the *Drosophila *E-APC protein has also been shown to localise to sites of cell-cell adhesion via its armadillo repeats [9]. Mislocalization of E-APC leads to the disruption or impairment of intercellular adhesion, implicating E-APC in the regulation of cell contacts in *Drosophila*. In the light of our study and recent observations by other investigators [8] it seems possible that APC might play a similar role in mammalian epithelial tissues.

Our observations of cells where both junctional and microtubule-associated GFP-APC populations were present indicated that these different pools did not spatially overlap. The question of how this might be achieved has parallels with a long-standing problem in APC biology: how is APC targeted to the tips of a subset of microtubule ends at specific sites at the cell periphery? Recent work from other investigators has indicated that an APC localisation to microtubule ends near free cell edges arises from a Cdc42-initiated signalling cascade that results in a local inhibition of GSK3β activity [28]. Phosphorylation by GSK3β inhibits the microtubule-binding ability of APC [22]. Therefore, local inhibition of the activity of this enzyme promotes the association of APC with microtubules in specific cellular regions where Cdc42 is active. Implicit in this model is the assumption that GSK3β activity elsewhere in the cell normally suppresses the association of APC with microtubules. If this model is combined with the observation that APC can localise to cell junctions when they are available then a plausible mechanism for generating the compartmentalisation seen in our study can be formulated.

## Conclusion

In this study we show that APC is capable to localising to both microtubules and to junctions within the cell, depending on cellular context. The junctional localisation is likely to be mediated by the N-terminal region of the APC protein. Consistent with this we find that colon cancer cell lines expressing truncated APC proteins are also capable of localising to the cortex in cells having the necessary cell-cell contacts. As well as losing function within the WNT signalling pathway, it seems possible that truncated APC proteins might act as dominant-negative mutants in cells that retain a normal copy of the APC protein, with the mutant copy interfering with the action of full-length APC molecules at intercellular junctions. This may have direct implications for the development of colorectal cancer.

## Methods

### Cells

COS-7 cells were cultured as described previously [29]. Caco-2 and SW480 cells were obtained from the Cancer Research UK Cell Line Service at the London Research Institute, Lincoln's Inn Fields, UK and cultured according to the instructions supplied. Incubations with cytoskeletal poisons were performed as described previously [29]. Nocodazole and Cytochalasin D were obtained from Sigma.

### Antibodies

The rabbit polyclonal anti M-APC antibody [3] was a kind gift from Dr Inke Näthke, (University of Dundee), and was used at a 1/500 dilution for immunostaining. The monoclonal APC antibody ALI 12-28 used in this study was obtained from Cancer Research UK Antibody Service at the London Research Institute and was used at a 1/500 dilution for immunostaining. It has also been made commercially available by Abcam and this antibody was used for Westerns at 1/5000 dilution. Rabbit polyclonal and mouse monoclonal anti-GFP antibodies were obtained from Clontech and used at a 1/1000 dilution for immunostaining and a 1/2000 dilution for Western Blotting. Rabbit polyclonal α-catenin, mouse monoclonal β-catenin, rabbit polyclonal pan-cadherin and mouse monoclonal β-actin antibodies were all obtained from Sigma. All secondary antibodies were Alexa 488 and 594 conjugates obtained from Molecular Probes, as was Alexa 594 conjugated phalloidin.

### Western Blotting

For Western blotting, cells were resuspended in RIPA buffer (50 mM Tris pH7.5, 150 mM NaCl, 1% (v/v) Igepal, 0.5% (v/v) Sodium Deoxycholate, 1 mM EDTA, 0.1% (v/v) SDS) buffer containing EDTA free complete protease inhibitors (Roche) and phosphastase inhibitors cocktail II (Sigma). Protein concentrations for the cell extracts was determined using a protein assay kit (Pierce). NuPage™ Loading Buffer (Invitrogen) was then added to 10–20 μg of protein extract following the manufacturers instructions. Proteins were then separated by SDS-PAGE using a 3–8% Tris-Acetate NuPage™ gradient gel system (Invitrogen). Proteins were transferred onto nitrocellulose membranes using NuPage™ transfer buffer as per manufacturers instructions (Invitrogen). After transfer the nitrocellulose membranes were incubated in 3% (w/v) BSA/PBS/0.1% (w/v) Tween 20 for 1 hour at room temperature. Membranes were then incubated overnight at 4°C with the specific antibody diluted in 3% (w/v) BSA/PBS/0.1% (w/v) Tween 20. After washes in PBS/0.1% Tween 20, membranes were incubated with an HRP-conjugated secondary antibody for 2 h before further extensive washing. Immunoreactivity was detected using the SuperSignal^® ^West Pico Chemiluminescent Substrate Kit (Pierce).

### Immunoprecipitation

For immunoprecipitation, COS-7 cells were transfected as described below (see Live Imaging) then resuspended in modified RIPA buffer (50 mM Tris pH7.4, 80 mM KCl, 10 mM EDTA, 1% Triton X-100 containing EDTA-free complete protease inhibitors (Roche) and phosphastase inhibitors cocktail II (Sigma)). Extracts were then immunoprecipitated with 2 μg of polyclonal anti-GFP antibody (Clontech) overnight at 4°C. The following day 40 μl of an 80% protein-G sepharose/PBS solution was added to each extract and incubated for a further 3 hours at 4°C. Extracts were then washed ×3 in modified RIPA buffer and the pellets resuspended in 30 μl PBS. Extracts were then processed for SDS-PAGE and Western blotting as described previously.

### Immunofluorescence

Cells were cultured on sterile coverglasses, processed for immunocytochemistry using cold methanol [29] or paraformaldehyde fixation [30] and imaged using the CCD camera based system described below in conjunction with excitation/emission filtersets for DAPI, FITC and TRITC.

### Live Imaging

Cells were grown and transfected in 35 mm glass-bottomed culture dishes (Iwaki brand; Asahi Techno Glass Corporation, Japan) obtained from Bibby Sterilin. Transfections were performed using GeneJuice (Novagen) according to the manufacturers instructions. 12–18 h after transfection the standard cell culture medium was replaced by 2 ml of pre-warmed medium supplemented with 20 mM HEPES. The cells were transferred to a Zeiss Axiovert 200 inverted microscope with a heated chamber enclosing the microscope stage (Solent Scientific, UK) allowing the temperature to be maintained at 37°C throughout imaging. Cells were imaged by fluorescence microscopy using a Zeiss Plan Apo 63X/1.4NA oil immersion lens. Time-lapse images were captured at 3–10s intervals for durations of 5–10 min using Ludl shutters and a Hamamatsu Orca II ER camera. Images were obtained using 2 × 2 binning with exposure times of less than 350 ms/frame. An excitation/emission filterset optimised for EGFP imaging was used (Chroma Technology Corp., Brattleboro, USA; filterset ID 86007). Microscope, camera, filterwheels and shutters were controlled by Kinetic Imaging AQM 6 software (Kinetic Imaging, Nottingham, UK). Cells expressing as low a level of fusion protein as could be successfully imaged using our CCD camera were used in this work. Brightly fluorescent cells, typically those clearly visible without the use of the camera, often displayed evidence of extensive microtubule bundling and were excluded from the study. Time-lapse image series were saved as uncompressed AVI files then cropped, compressed and converted into Quicktime movies using Adobe ImageReady CS. Tracking analyses were performed on unprocessed data files using Motion Analysis software from Kinetic Imaging.

### Expression constructs

The pEGFP-APC plasmid used in this study was a kind gift from Dr. J. Victor Small (Salzburg, Austria). It directs the expression of full length human APC (2843aa) *N*-terminally tagged with the fluorescent protein eGFP. This construct was subjected to a range of restriction digests and sequencing to confirm its identity. This indicated that the plasmid directs the expression of a full-length GFP-APC protein (Figure 1, panel A) similar to that previously described [31] rather than a N-terminally truncated protein as recently used by other workers [11]. A further four constructs were produced by restriction enzyme digestion of this plasmid. The first of these, pEGFP-APCΔC, was obtained by digestion of pEGFP-APC with *BspE*I and *Avr*II, resulting in an APC fragment lacking its final 54 amino acids. This fragment was ligated into pEGFP-C1 (Clontech) digested with *Kpn*I and *Xba*I (Figure 1, panel B). A second construct, pEGFP-APCΔNΔC, was obtained by digestion of pEGFP-APC with *Kpn*I and *Avr*II. This results in the removal of the first 206 amino acids from the *N*-terminus of APC in addition to the removal of the last 54 amino acids. This product was cloned into pEGFP-C1 digested with *BspE*I and *Xba*I (Figure 1, panel C). The third construct, pEGFP-APC-C, was created by PCR amplification of a *C*-terminal APC fragment using the following primers to obtain the last 170 amino acids of APC:

GFPC1For - 5'CCTAGATCTTCCGGATCTCCCACAG3'

GFPC1Rev -5'TTAGTTTCATGGTACCTCTCTTTTA3'

The forward primer contains a *BspE*I restriction site and the reverse primer a *Kpn*I restriction site to allow subcloning of the PCR product into pEGFP-C1 digested with the same enzymes (Figure 1, panel D). A fourth construct, pEGFP-APC-N, was obtained by digesting pEGFP-APC with *BspE*I and *Hind*IIII and cloning the resulting product into pEGFP-C1. The resulting plasmid directs the expression of the first 746 amino acids of APC *N*-terminally fused to EGFP (Figure 1, panel E).

## Authors' contributions

KJM contributed to the design of the study, the construction of GFP expression vectors, live cell imaging, western blotting, immunostaining and drafted the manuscript. JMA contributed reagents and helped to revise the manuscript. TL maintained cell cultures and helped revise the manuscript. EEM conceived the study, participated in its design and execution and helped in the drafting and revision of the manuscript. All authors read and approved the final manuscript

## Supplementary Material

Additional File 1 Microtubule-dependent localisation of full-length GFP-APC expressed in subconfluent COS-7 cells. This movie shows a subconfluent cell with GFP-APC associated with microtubule tips at specific regions near the cell periphery, typically in cell vertices. Microtubule tip labelling was comprised of discrete GFP-APC puncta or clusters in conjunction with a dimmer, less specific labelling of microtubule distal segments. Image capture rate was 1frame/5s using a 63× oil immersion lens. Movie is played back at 20 frames/s. Click here for file

Additional File 2 Peripheral deposition of GFP-APC puncta in COS-7 cells. Microtubule-associated GFP-APC puncta can be deposited at the cell periphery by shrinking microtubules. Two microtubules can be seen to grow towards the cell periphery depositing GFP-APC puncta at the cortex (arrowheads). Once the puncta has contacted the cell periphery the microtubule can be seen to shrink away from the cell edge leaving the original puncta at the periphery. Close observation of this image sequence suggested that GFP-APC puncta might remain associated with shrinking microtubule ends; this is examined in more detail in subsequent movies. Image capture rate was 1 frame/5s using a 63× oil immersion lens and the movie is played back at 20 frames/s. Click here for file

Additional File 3 Retrograde movement of GFP-APC puncta in COS-7 cells. Peripheral GFP-APC puncta underwent linear retrograde movements with an average velocity of 21.6 ± 2.4 μm. An example is indicated in this movie by the arrowhead. These movements predominantly occurred over short distances of less than 10 μm. Image capture rate was 1 frame/3s using a 63× oil immersion lens. The movie is played back at 10 frames/s. Click here for file

Additional File 4 Partial deposition of microtubule-associated GFP-APC puncta in COS-7 cells. The movie shows a GFP-APC puncta (arrowhead) undergoing retrograde movement on a shrinking microtubule end within the cytoplasm. Part of this original puncta is deposited within the cytoplasm (arrowhead 2) while the remainder continues retrograde movement (arrowhead 1). Note that close examination of additional files 2 and 3 reveal similar behaviours. Image capture rate was 1 frame/5s using a 63× oil immersion lens. The movie is played back at 10 frames/s. Click here for file

Additional File 5 Microtubules possessing GFP-APC puncta could be seen to undergo shrinkage followed by a period of pause and then growth (arrowhead). GFP-APC puncta remained at the end of the microtubule throughout this cycle of shrinkage and growth. Of interest in this sequence is the observation that separate microtubule-associated GFP-APC puncta coalesce into a single large structure at the microtubule tip during shrinkage, then separate out into a distribution resembling beads on a string during microtubule regrowth, before beginning to re-coalesce on the paused microtubule tip at the cell periphery. Image capture was 1 frame/5s using a 63× oil immersion lens, the movie is played back at 10 frames/s. Click here for file

Additional File 6 Comet-like GFP-APC expression in COS-7 cells. Occasionally cells were found with a motile comet-like distribution within the cell interior. These comets moved with an average velocity of 20.4 ± 6 μm/min, essentially indistinguishable from the distribution and behaviour observed for the microtubule tip-tracking APC ligands EB1 and EB3 when expressed as GFP fusion proteins in COS-7 cells. Image capture rate was 1 frame/3s using a 63× oil immersion lens and movie is played back at 20 frames/s. Click here for file

Additional File 7 Fast moving GFP-APC puncta within the cytoplasm of COS-7 cells. GFP-APC puncta were occasionally observed undergoing rapid transport within the cell interior at velocities in the region of 2 μm/s, speeds consistent with microtubule motor-mediated transport. Microtubule and junction-associated GFP-APC populations are also visible at the cell periphery in this sequence. Image capture rate was 1 frame/3s using a 63× oil immersion lens and movie is played back at 20 frames/s. Click here for file

Additional File 8 Cortically-localised GFP-APC in COS-7 cells. In confluent cultures of transfected COS-7 cells GFP-APC was present as a discontinuous array of punctate structures associated with membranes contacting adjacent cells. These puncta were far less dynamic than microtubule-associated GFP-APC distributions, being essentially immobile relative to the cell cortex. Image capture rate was 1 frame/10s using a 63× oil immersion lens and movie is played back at 20 frames/s. Click here for file

Additional File 9 Regional specificity of different GFP-APC localisations in COS-7 cells. In partially confluent COS-7 cells having both contacted and free edges, microtubule and junction-associated GFP-APC populations exist in the same cell but never overlap. Image capture was rate 1 frame/5s using a 63× oil immersion lens and movie is played back at 20 frames/s. Click here for file

Additional File 10 Live imaging of Nocodazole treated COS-7 cell expressing GFP-APC. This movie shows a cell with junction-associated GFP-APC (arrowheads) and microtubule-associated GFP-APC (arrow). After addition of Nocodazole to a final concentration of 5 μg/ml the microtubule-associated GFP-APC population is rapidly lost whereas the junction-associated GFP-APC is largely unaffected. Image capture rate was 1 frame/10s and movie played back at 20 frames/s. Click here for file

Additional File 11 Live imaging of Cytochalasin D treated COS-7 cell expressing GFP-actin. This movie shows two cells forming cell-cell contacts. A cortical actin belt can be seen around the circumference of the cells. Upon addition of 1 μg/ml Cytochalasin D the cortical actin belt is weakened and contracts along the cell edge (arrowheads). Image capture rate was 1 frame/min, movie played back at 20 frames/s. Click here for file

Additional File 12 Live imaging of Cytochalasin D treated COS-7 cell expressing GFP-APC. This movie shows two GFP-APC expressing cells forming a cell-cell contact. GFP-APC is present along the cortex at the site of cell-cell contact and on microtubules near a free edge in the upper cell. Upon addition of Cytochalasin D the cell-cell contact between the cells is weakened and undergoes breakage (black arrow). The cortical GFP-APC puncta are then seen to contract towards the cell vertices in the direction of the white arrow. The microtubule-associated GFP-APC appears unaffected by Cytochalasin D treatment, within the constraints imposed by retraction of the cell edge. Image capture rate was 1 frame/min, movie played back at 20 frames/s. Click here for file

Additional File 13 Expression of the short N-terminal APC construct led to the identification of 3 distinct localisations. The first was to the centrosome (arrow), the second to small, sometimes motile puncta within the cytoplasm, and the final localisation was to sites of cell-cell adhesion (arrowheads). Image capture rate was 1 frame/10s using a 63× oil immersion lens and the movie is played back at 20 frames/s. Click here for file
